# Genome Sequence of the Emerging Mycotoxin-Producing Filamentous Fungus Fusarium tricinctum Strain INRA104

**DOI:** 10.1128/genomeA.00509-18

**Published:** 2018-06-21

**Authors:** Nadia Ponts, Florence Richard-Forget, Honghai Zhang, Gérard Barroso, Chen Zhao

**Affiliations:** a INRA UR1264, MycSA, Villenave d’Ornon, France; b Academy of State Administration of Grain, Beijing, China; c Bordeaux University, Bordeaux, France

## Abstract

The genome of the phytopathogenic fungus Fusarium tricinctum strain INRA104 was sequenced at a fold-coverage of more than 500×. This led to 23 scaffolds, including one scaffold for the mitochondrial genome, for a total genome size of 42.8 Mb, with an average GC content of 45% and 13,387 predicted genes.

## GENOME ANNOUNCEMENT

Up to 15 Fusarium spp. can be found on cereal ears with Fusarium head blight (FHB) symptoms (1). Most of these fungal species are able to produce several types of mycotoxins that first accumulate in grains and then contaminate processed products in the food chain, representing both a health risk and an important economic stake. Some of these fusariotoxins are already subject to international regulation (deoxynivalenol, fumonisins, and zearalenone). In addition, other mycotoxins are considered emerging because they were recently found in crops in Europe and Asia (2) and, by their suspected toxicity, constitute subjects of interest for food security agencies. Regulations setting threshold values for contamination should be published shortly. Indeed, emerging mycotoxins, such as enniatins, beauvericin, and moniliformin, have been reported to possess genotoxic effects *in vitro* (3). Enniatin B1 and beauvericin could be more hepatotoxic than major and regulated toxins, such as aflatoxin B1 (4). Despite these significant potential health risks, there are still few data on enniatins, including the regulation of their biosynthesis and their secretion by the producing species. Enniatins are cyclohexadepsipeptides synthesized outside the ribosome by multifunctional enzymes, including enniatin synthase (ESyn1, 347 kDa) (5). However, the characterization of the different genes encoding and regulating the enzymes involved in the production of the 29 enniatins described to date is still a challenge (5). On cereals, the enniatins are produced by Fusarium avenaceum, for which three reference genomes are available (6), and Fusarium tricinctum. In this context, the genome of an enniatin- and other-mycotoxin-producing strain of F. tricinctum was sequenced.

The F. tricinctum strain INRA104 was isolated in 2001 from corn kernels collected in a French field from an agricultural region located roughly 150 km south of Paris (i.e., department number 45, Loiret). Genomic DNA was extracted from freeze-dried mycelia (1 week of liquid culture in glucose-yeast extract-neopeptone [GYEP] medium) using the cetyltrimethylammonium bromide (CTAB) method (7). A combination of third-generation sequencing (PacBio Sequel platform) and next-generation sequencing (Illumina HiSeq platform) produced more than 22 billion bases. After read correction (FALCON pipeline v1.8.8-1 [PacBio]), assembly (SMART *de novo*, for the nuclear genome, and an in-house modified version of CANU v1.7 [8] for the mitochondrial genome), and genome polishing (GenomicConsensus package [PacBio] and Pilon v1.22 [9]), we obtained 22 scaffolds for a genome size of 42.8 Mb (with sizes ranging from 7,333 bp to 5,080,745 bp; mean size, 1,782,168 bp; *N*_50_ value, 2,710,832 bp; *N*_90_ value, 1,246,557 bp; GC content, 45%) plus one unique scaffold (GenBank accession number CM009895) for the mitochondrial genome, consisting of 48,506 bp (GC content, 33%). *De novo* gene prediction using the genome of Fusarium graminearum PH-1 as a reference (AUGUSTUS v3.3 [10]) identified 13,387 protein-coding genes, with an average length of 1.7 kbp.

### Accession number(s).

This whole-genome shotgun project has been deposited at DDBJ/ENA/GenBank under the accession number QFZF00000000. The version described in this paper is version QFZF01000000.

## References

[1] Beccari G, Prodi A, Tini F, Bonciarelli U, Onofri A, Oueslati S, Limayma M, Covarelli L 2017 Changes in the *Fusarium* head blight complex of malting barley in a three-year field experiment in Italy. Toxins 9:120. doi:10.3390/toxins9040120. PMC5408194 28353653

[2] Jestoi M 2008 Emerging *Fusarium*-mycotoxins fusaproliferin, beauvericin, enniatins, and moniliformin—a review. Crit Rev Food Sci Nutr 48:21–49. doi:10.1080/10408390601062021. 18274964

[3] Fraeyman S, Croubels S, Devreese M, Antonissen G 2017 Emerging *Fusarium* and *Alternaria* mycotoxins: occurrence, toxicity and toxicokinetics. Toxins 9:228. doi:10.3390/toxins9070228. PMC5535175 28718805

[4] Svingen T, Lund Hansen N, Taxvig C, Vinggaard AM, Jensen U, Have Rasmussen P 2017 Enniatin B and beauvericin are common in Danish cereals and show high hepatotoxicity on a high-content imaging platform. Environ Toxicol 32:1658–1664. doi:10.1002/tox.22367. 27628925

[5] Sy-Cordero AA, Pearce CJ, Oberlies NH 2012 Revisiting the enniatins: a review of their isolation, biosynthesis, structure determination and biological activities. J Antibiot 65:541–549. doi:10.1038/ja.2012.71. 22990381 PMC3573854

[6] Lysøe E, Harris LJ, Walkowiak S, Subramaniam R, Divon HH, Riiser ES, Llorens C, Gabaldón T, Kistler HC, Jonkers W, Kolseth A-K, Nielsen KF, Thrane U, Frandsen RJN 2014 The genome of the generalist plant pathogen *Fusarium avenaceum* is enriched with genes involved in redox, signaling and secondary metabolism. PLoS One 9:e112703. doi:10.1371/journal.pone.0112703. 25409087 PMC4237347

[7] Noël T, Labarère J 1989 Isolation of DNA from *Agrocybe aegerita* for the construction of a genomic library in *Escherichia coli*. Mushroom Sci 1:187–201.

[8] Koren S, Walenz BP, Berlin K, Miller JR, Bergman NH, Phillippy AM 2017 Canu: scalable and accurate long-read assembly via adaptive *k*-mer weighting and repeat separation. Genome Res 27:722–736. doi:10.1101/gr.215087.116. 28298431 PMC5411767

[9] Walker BJ, Abeel T, Shea T, Priest M, Abouelliel A, Sakthikumar S, Cuomo CA, Zeng Q, Wortman J, Young SK, Earl AM 2014 Pilon: an integrated tool for comprehensive microbial variant detection and genome assembly improvement. PLoS One 9:e112963. doi:10.1371/journal.pone.0112963. 25409509 PMC4237348

[10] Stanke M, Steinkamp R, Waack S, Morgenstern B 2004 AUGUSTUS: a Web server for gene finding in eukaryotes. Nucleic Acids Res 32:W309–W312. doi:10.1093/nar/gkh379. 15215400 PMC441517

